# Diabetes mellitus in a young Amazon Indian child

**DOI:** 10.1590/S1516-31802005000200012

**Published:** 2005-03-02

**Authors:** Mônica Andrade Lima Gabbay, Edson Bussad, Ligia Persoli, Walkiria Volpini, Sérgio Atala Dib

**Keywords:** Diabetes Mellitus, Ethnic Groups, HLA antigens, South american indians, Autoantibodies, C-peptide, Diabetes Mellitus, Índio, Antígenos HLA, Auto-anticorpos, Peptídeo C, Grupos étnicos, Índios sul-americanos

## Abstract

**CONTEXT:**

Although type 2 diabetes has been described among American Indian children, no case of type 1 diabetes has been reported in the literature.

**CASE REPORT:**

We report the first case of diabetes in a South American Indian child from the tropical rainforest, who was positive for IA2 autoantibodies and genetic markers of susceptibility to type 1 diabetes, but also demonstrated residual beta cell function four years after diagnosis.

## RESUMO

**CONTEXTO:**

O diabetes *mellitus* tipo 2 tem sido descrito em populações indígenas, mas casos de diabetes *mellitus* tipo 1 são raros e muitas vezes não bem caracterizados, devido às condições inerentes ao meio em que habitam os índios.

**RELATO DE CASO:**

Nós relatamos aqui o primeiro caso de diabetes do tipo 1 em uma criança indígena da América do Sul, proveniente da floresta Amazônica, caracterizado por marcadores genéticos, imunológicos e clínicos, mas que apresentava função residual das células beta após quatro anos do diagnóstico, o que pode dificultar, às vezes, a caracterização clínica do tipo especifico de diabetes em algumas populações.

## INTRODUCTION

Type 1 diabetes mellitus (T1DM) is still considered to be the commoner form of diabetes mellitus in young people. However, T1DM has not been reported in the literature in any child or adolescent known to have full American Indian heritage. None of the probable case subjects with type 1 diabetes described in a recent study^1^ population were known to be of full American Indian heritage^2^.

We have recently reported^3^ on latent autoimmune diabetes of the adult (LADA) in a Xavante-Jê Indian from the western groups of the Sangradouro community in Poxoréu, State of Mato Grosso, Brazil. Here, we show the first case of diabetes in a South American Indian child from the Yanomami reserve in the tropical rainforest of northern Brazil and southern Venezuela.

## CASE REPORT

A 6-year-old Yanomami Indian girl was referred to the Diabetes Center of Universi-dade Federal de São Paulo (Unifesp) to control her diabetes. This disease was diagnosed at the age of two years, during treatment for a tuberculosis infection. At that time, she developed fatigue, thirst, polyuria, weight loss and fasting blood glucose (FBG) of 480 mg/dl. Following this, she was given the recommendation to have a daily shot of insulin (0.5 units/kg of body weight), but her family refused to give her the shots, and so this therapy became irregular. Over the last three years, she has presented three diabetic ketoacidosis crises. She is the ninth daughter of short-stature parents. There is no history of diabetes mellitus in her family.

When she was first seen at the Diabetes Center, her physical examination was normal, despite her height of only 92 cm (-3.7 standard deviations, SD) and weight of only 12.1 kg (-2.1 SD). She had FBG of 510 mg/dl, negative ketonuria, HbA1c [glycated haemoglobin of 7.8% (normal value, nv: 2.5-5.5%), normal albuminuria and normal retinal examination. Autoantibodies directed against glutamic acid decarboxylase (GAD-65) and tyrosine phosphatase-related enzyme proteins (ICA512/IA2) were measured using quantitative radiobinding assays. Anti-GAD65 was negative on two different occasions (GAD 65Ab index: 0.21 and −0.02 U; nv < 0.22U) and the ICA512/IA2 Ab index was 3.71 U/ml (nv < 0.97 U/ml). At this time, the patient's fasting serum C-peptide (FCP) level, measured by radioimmunoassay, was 0.64 pmol/ml (nv: 0.11-1.2 pmol/ml). Her human leukocyte antigen (HLA) typing showed DRB1*0411, DQA1*0303, DQB1*0302/DRB1*14, DQA1*0503 and DQB1*0307. These haplotypes were identical to those of her parents.

Over a two-week period, while her parents were attending a diabetes education program, her glycemic levels became normalized by means of human pre-mixture (80N/20R) insulin before breakfast and supper (0.9 units/kg/day). She then went back home to the Yanomami Indian reserve.

## DISCUSSION

There are twelve thousand Yanomami Indians alive today^4^, in about 230 villages, with a population of around 150-200 individuals in each village (95% of them inside the Amazon forest and the others along the major rivers).^4^ They have had contact with the civilized world only over the last 20 years.^4^ Their diet (sweet potatoes, vegetables and bananas) is monotonous. Their most common illnesses are malaria, infectious diarrhea and acute respiratory diseases.^4^

Bloch et al., studying an Indian adult population from this group, found higher mean capillary blood glucose among women than among men, although still within the normal range. These blood glucose levels correlated with the body mass index and waist-to-hip circumference ratio.^5^

The diabetic Yanomami child reported on here expressed the DRB1*04-DQB1*0302 haplotype that is associated with susceptibility to type 1 diabetes in Caucasians and also in our mixed Brazilian population.^6^ Furthermore, we found a great diversity of DRB1*04 alleles in Brazilian families with type 1 diabetes in our previous study.

Her FCP (0.64 pmol/ml) was higher than the Diabetes Control and Complication Trial (DCCT) criterion (less than 0.2 pmol/ml) to insulin-dependent diabetes mellitus (IDDM).^7^ This FCP level suggests that she still had residual beta cell function four years after the clinical diagnosis. The absence of GAD65 antibodies in this girl could be due to antibodies becoming seronegative during disease evolution or it could suggest Type 1B diabetes. It is interesting that, some years ago, we reported^8^ on a case of young Indian man from another group (Krenak-Macro-Jê) whose diabetes was diagnosed by means of spontaneous ketoacidosis, but who did not have autoantibodies (anti-insulin and islet beta cells) and had a normal FCP (0.95 pmol/ml). Therefore, both cases could be classified initially as atypical diabetes. Recently, the metabolic and immunological features of Chinese patients with atypical diabetes mellitus has also been described.^9^

Idiopathic type 1 diabetes, by definition, has no known etiology. Individuals with this form of diabetes can have heterogeneous clinical manifestations. In African-American patients, it has been called atypical diabetes or flatbush diabetes. These individuals can have diabetic ketoacidosis as their initial clinical presentation, but autoimmune islet cell markers are absent at diagnosis and they have physical characteristics similar to type 2 diabetes patients.^10^

Asian idiopathic type 1 diabetes cases have different features. Urakami et al.^11^ reported, among 85 Japanese children with type 1 diabetes, that 16.5% had a new subtype of type 1 diabetes that was characterized by fulminant onset, absolute insulin deficiency and absence of beta-cell autoimmunity markers. These patients had high-risk HLA typing (either HLA-DR4 or DR9) and low or undetectable serum C-peptide values.

Harwell et al.,^12^ studying a young American Indian population (aged < 20 years) from Montana and Wyoming (United States), considered that children with diabetes had type 1 diabetes if aged ≤ 5 years, with weight-for-age lower than the 15^th^ percentile at diagnosis or positive results from islet cell antibody tests less than one year after diagnosis. They considered children to have probable type 2 diabetes if their weight-for-age was ≥ 85^th^ percentile, acanthosis nigricans was noted, serum C-peptide or insulin was high within one year of diagnosis, there was a family history of type 2 diabetes, oral hypoglycemic agents with or without insulin were used at follow-up more than one year after diagnosis, or there was no current pharmacological treatment one year after diagnosis.

In accordance with these criteria our diabetic Indian child had the characteristics of type 1 (age less than 5 years at diagnosis, low body weight, positive for IA2 antibodies, genetic markers for type 1 diabetes and in need of insulin treatment since diagnosis). Nonetheless, she still had residual endogenous insulin secretion, as shown by FCP levels.

In summary, this Amazon Indian emphasizes the difficulty in accurately classifying diabetes by type among young people from the same ethnic groups.

Population-based studies that characterize both residual beta-cell function and multiple autoimmune antibodies serially are needed to define the natural history of diabetes and clarify the potential heterogeneity of the disease in young Indian populations.
